# Exploring the Interactions of Ruthenium (II) Carbosilane Metallodendrimers and Precursors with Model Cell Membranes through a Dual Spin-Label Spin-Probe Technique Using EPR

**DOI:** 10.3390/biom9100540

**Published:** 2019-09-27

**Authors:** Riccardo Carloni, Natalia Sanz del Olmo, Paula Ortega, Alberto Fattori, Rafael Gómez, Maria Francesca Ottaviani, Sandra García-Gallego, Michela Cangiotti, F. Javier de la Mata

**Affiliations:** 1 Department of Pure and Applied Sciences, University of Urbino “Carlo Bo”, 61029 Urbino, Italy; riccardocarloni93@gmail.com (R.C.); alberto.fattori@uniurb.it (A.F.); maria.ottaviani@uniurb.it (M.F.O.); 2 Department of Organic and Inorganic Chemistry, and Research Institute in Chemistry “Andrés M. del Río” (IQAR), University of Alcalá, 28805 Madrid, Spainpaula.ortega@uah.es (P.O.); rafael.gomez@uah.es (R.G.); sandra.garciagallego@uah.es (S.G.-G.); 3 Networking Research Center on Bioengineering, Biomaterials and Nanomedicine (CIBER-BBN), 088034 Barcelona, Spain; 4 Institute Ramón y Cajal for Health Research (IRYCIS), 28034 Madrid, Spain

**Keywords:** electron paramagnetic resonance, dendrimer, metallodendrimer, ruthenium, cell membrane, spin probe, cancer

## Abstract

Dendrimers exhibit unique interactions with cell membranes, arising from their nanometric size and high surface area. To a great extent, these interactions define their biological activity and can be reported in situ by spin-labelling techniques. Schiff-base carbosilane ruthenium (II) metallodendrimers are promising antitumor agents with a mechanism of action yet to explore. In order to study their in situ interactions with model cell membranes occurring at a molecular level, namely cetyltrimethylammonium bromide micelles (CTAB) and lecithin liposomes (LEC), electron paramagnetic resonance (EPR) was selected. Both a spin probe, 4-(*N*,*N*-dimethyl-*N*-dodecyl)ammonium-2,2,6,6-tetramethylpiperidine-1-oxyl bromide (CAT12), able to enter the model membranes, and a spin label, 2,2,6,6-tetramethylpiperidine-1-oxyl (TEMPO) covalently attached at newly synthesized heterofunctional dendrimers, were used to provide complementary information on the dendrimer–membrane interactions. The computer-aided EPR analysis demonstrated a good agreement between the results obtained for the spin probe and spin label experiments. Both points of view suggested the partial insertion of the dendrimer surface groups into the surfactant aggregates, mainly CTAB micelles, and the occurrence of both polar and hydrophobic interactions, while dendrimer–LEC interactions involved more polar interactions between surface groups. We found out that subtle changes in the dendrimer structure greatly modified their interacting abilities and, subsequently, their anticancer activity.

## 1. Introduction

Current anticancer therapies are poorly efficient because of the non-specific drug distribution, the development of multidrug resistance, and the intrinsic heterogeneity of cancer. In the cancer field, and in many other fields, nanotechnology provides new tools that increase the therapeutic efficiency and minimize side-effects. Nanotechnology provides multifunctional platforms to be used either as therapeutic agents, as drug delivery systems [1,2], or as contrast agents with longer circulation time for the early detection and diagnosis of diseases [3]. These multifunctional platforms may further be designed to simultaneously present two or more activities in a single molecule, the so-called nanotheranostics. Nanotheranostics are cutting-edge nanoparticles (NPs) designed for simultaneously providing accurate diagnosis together with effective therapeutic activity, monitoring drug release and distribution in real time, and have been already used in different fields including cancer [4,5] and neurological disorders [6]. The different nanotheranostic agents reported in the literature [7]—metal nanoparticles, polymeric nanoparticles, and dendrimers—are useful tools for optimizing treatment outcomes in cancer and other diseases.

In particular, dendrimers are excellent platforms for accurate heterofunctionalization. They are the flagship of precision nanoparticles, whose monodisperse scaffold enables a controlled design of the groups located at the periphery or within the interior [8]. They allow a precise structure-to-activity relationship, unattainable by other nanoparticles, exhibiting different biological activity depending on both their functional groups and the nature of their scaffold. As an example, carbosilane dendrimers rely on a hydrophobic scaffold, comprising multiple C–C and C–Si bonds, which provide extraordinary flexibility and stability and enhance the interaction with biological membranes. Many biomedical applications have been reported for this dendritic family, such as antiviral and antibacterial agents, non-viral vectors for nucleic acid delivery, and antitumor drugs, among others [9].

Dendrimers interact well with membranes and cells [10,11], and thus trigger multiple biological activities. The nanoparticle–cell interactions are modulated by both the physicochemical properties of the NPs and cell-specific parameters [12], and a thorough understanding of this interaction can provide valuable information for the design of effective drugs. Among the available tools for studying nanoparticle–cell interactions, electron paramagnetic resonance (EPR) spectroscopy has excelled in providing on-site structural and dynamical information. The spin-probe/spin-label-based EPR technique has already been demonstrated to be a powerful tool in characterizing the interactions of dendrimers with model membranes and cells [10,13,14,15,16,17,18,19,20,21,22,23,24], and has even been used in living systems [25]. Importantly, EPR spectroscopy shares many of the features of magnetic resonance imaging (MRI), including the underlying principles, and exhibits superior detection sensitivity. The recent developments in EPR instrumentation, probes, and methods offer opportunities in potential areas of clinical application [26], such as oximetry and dosimetry [27].

We have recently reported the use of Schiff-base containing carbosilane dendrimers as efficient chelators of copper (II) [28,29] and ruthenium (II) [30,31] complexes. The inclusion of metallodrugs in nanostructures improves their delivery and penetration—mainly by endocytosis—thus increasing the concentration in cells and subsequently the anticancer effect [32]. Accordingly, the carbosilane metallodendrimers exhibited promising antitumor activity in both in vitro and in vivo assays. Further insight into the antitumor activity of the Cu(II) metallodendrimers was obtained through the EPR analysis of the dendrimers’ interaction with model membranes [29]. We found out that the dendritic generation, as well as the metal counter-ion, tuned the strength of interaction of the dendrimer with the membrane and affected the toxicity and selectivity towards cancer cells.

With the aim of obtaining more information about the interactions occurring at a molecular level in the biological environment of Schiff-base carbosilane dendrimers and their Ru(II) complexes, we selected the EPR technique to study the site–site interactions between the dendrimers and model cell membranes, namely cetyltrimethylammonium bromide micelles (indicated as CTAB) and lecithin liposomes (indicated as LEC). To characterize these interactions in situ, both a spin probe, 4-(*N*,*N*-dimethyl-*N*-dodecyl)-ammonium-2,2,6,6-tetramethylpiperidine-1-oxyl bromide (CAT12) able to enter the model membranes, and a spin label, 2,2,6,6-tetramethylpiperidine-1-oxyl (TEMPO) covalently attached to the dendrimer surface, were used to provide complementary information on the dendrimer–model membrane interactions from both sides. A comparative EPR analysis was performed on the labelled dendrimers in the absence and presence of the model membranes. Relevant insight was obtained related to the influence of different parameters—generation, equilibration time, peripheral groups, and presence of heterofunctional ligands—on the nanoparticle–membrane interactions, which can explain their biological activity.

## 2. Materials and Methods

### 2.1. Dendrimers and Metallodendrimers

**Figure 1:**
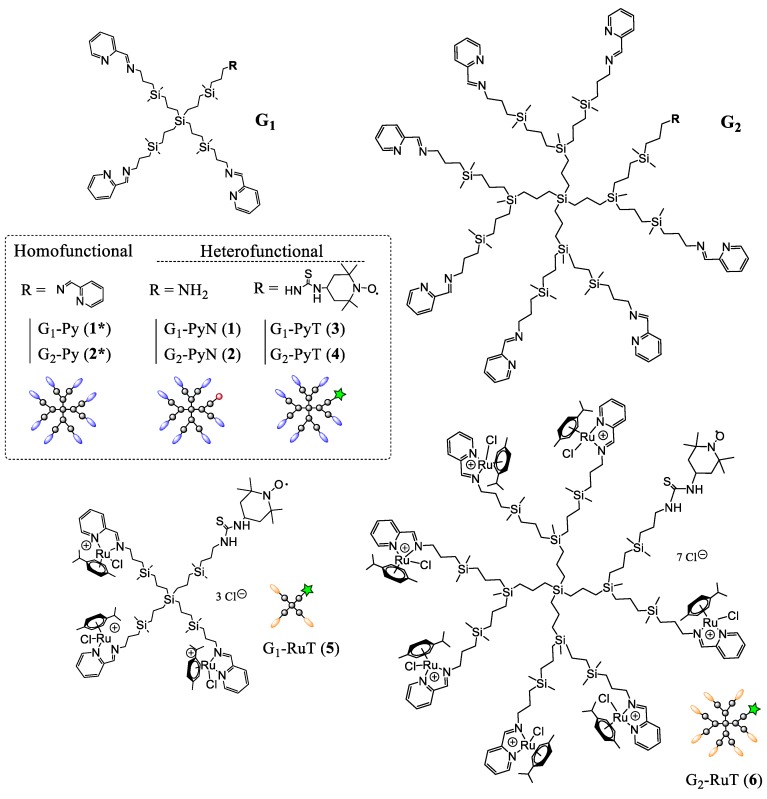
Structural representation of dendrimers and metallodendrimers used in the electron paramagnetic resonance (EPR) study. Homofunctional dendrimers: G_n_-Py (1*, 2*); heterofunctional dendrimers: G_n_-PyN (**1**,**2**) and G_n_-PyT (**3**,**4**); heterofunctional metallodendrimers: G_n_-RuT (**5**,**6**).

In order to evaluate the dendrimer–membrane interactions from the dendrimer point of view, novel labelled iminopyridine dendrimers were prepared (Figure 1). The three-step synthetic route included the preparation of the heterofunctional dendrimers G_n_-{[NCPh(*o*-N)]_m-1_[NH_2_]} [17] (for simplicity G_n_-PyN; n = 1, m = 4 (**1**); n = 2, m =8 (**2**)), G_n_-{[NCPh(*o*-N)]_m-1_[NHC(S)NH-TEMPO]} (G_n_-PyT, n = 1, m = 4 (**3**); n = 2, m = 8 (**4**)), and G_n_-{[NCPh(*o*-N)Ru(η^6^-*p*-cymene)Cl_2_]_m-1_[NHC(S)NH-TEMPO]} (G_n_-RuT; n = 1, m = 4 (**5**); n = 2, m = 8 (**6**)). The synthetic protocols and characterization details are described below for each dendrimer. 

For comparison, iminopyridine homofunctional dendrimers G_n_-{[NCPh(*o*-N)]_m_ (G_n_-Py; n = 1, m = 4 (1*); n = 2, m = 8 (2*) and the mononuclear counterpart n = 0, m = 1 (0*)) were used in EPR evaluation. Overall, these dendrimers provided information about the effect of dendritic generation (G_1_ vs. G_2_), the functional groups (G_n_-Py vs. G_n_-PyN), the labelling (G_n_-Py vs. G_n_-PyT) and the metal complexation (G_n_-PyT vs. G_n_-RuT), the latter known to enhance anticancer drug properties [32].

*G_1_-{[NCPh(*o*-N)]_3_[NH_2_]} (**G_1_-PyN**, **1**)*. To a solution of precursor G_1_-[NH_2_]_4_ (**I**) (313.1 mg, 0.47 mmol) in anhydrous tetrahydrofuran (THF), 2-pyridinecarboxaldehyde (152.1 mg, 1.42 mmol) was added. The mixture was stirred under inert atmosphere at room temperature in the presence of anhydrous MgSO_4_ for 12 h. Subsequently, the solution was filtered and the solvent was evaporated to obtain compound **1** as brown oil in quantitative yield. ^1^H-NMR (CDCl_3_): δ -0.06 (s, 24H, -(C*H_3_*)_2_Si-); 0.43 (m, 2H, (-C*H_2_*CH_2_CH_2_NH_2_); 0.51 (m, 24H, -SiC*H_2_*-); 1.29 (m, 8H, -SiCH_2_C*H_2_*CH_2_Si); 1.37 (m, 2H, (-SiCH_2_C*H_2_*CH_2_NH_2_); 1.68 (m, 6H, -SiCH_2_C*H_2_*CH_2_NCPh); 2.62 (m, 2H, (-C*H_2_*NH_2_); 3.62 (m, 6H,-C*H_2_*NCPh); 7.28 (m, 3H, Ar); 7.70 (m, 3H, Ar); 7.95 (m, 3H, Ar); 8.60 (m, 3H, Ar); 8.33 (s, 3H, -N=CH^imine^). ^13^C{^1^H}-NMR (CDCl_3_): δ -3.33 (-(*C*H_3_)_2_Si-); 1.00 (-(*C*H_3_)_2_SiCH_2_CH_2_CH_2_NH_2_); 12.1 (-Si*C*H_2_CH_2_CH_2_NH_2_); 13.1 (-Si*C*H_2_CH_2_CH_2_NCPh); 17.5, 18.5, 20.2 (-Si*C*H_2_*C*H_2_*C*H_2_Si-); 25.3 (-SiCH_2_*C*H_2_CH_2_NCPh); 28.3 (-SiCH_2_*C*H_2_CH_2_NH_2_); 45.5 (-*C*H_2_NH_2_); 52.9 (-*C*H_2_NCPh); 121.2, 124.5, 136.5, 149.3, 161.7 (CAr); 161.7 (-N=CH^imine^). Elemental analysis (%): calculated for C_50_H_89_N_7_Si_5_ (928.7 g/mol): C, 64.66; H, 9.66; N, 10.56; found: C, 64.30; H, 9.00; N, 10.48.

*G_2_-{[NCPh(*o*-N)]_7_[NH_2_]} (**G_2_-PyN**, **2**).* Dendrimer G_2_-PyN was prepared through the same procedure as compound **1,** using the following reagents: G_2_-[NH_2_]_8_ (**II**) (113.0 mg, 0.069 mmol), 2-pyridinecarboxaldehyde (51.73 mg, 0.483 mmol). Dendrimer **2** was isolated as brown oil in quantitative yield. ^1^H-NMR (CDCl_3_): δ -0.12 (s, 12H, -(C*H_3_*)Si(CH_2_CH_2_CH_2_Si)_2_); -0.06 (s, 48H, -(C*H_3_*)_2_Si-); 0.51 (m, 64H, -SiC*H_2_*-); 1.26 (m, 24H, -SiCH_2_C*H_2_*CH_2_Si- and -SiCH_2_C*H_2_*CH_2_NH_2_); 1.68 (m, 16H, (-SiCH_2_C*H_2_*CH_2_NCPh); 2.62 (m, 2H, (-C*H_2_*NH_2_); 3.63 (m, 14H,-C*H_2_*NCPh); 7.28 (m, 7H, Ar); 7.71 (m, 7H, Ar); 7.97 (m, 7H, Ar); 8.62 (m, 7H, Ar); 8.35 (s, 7H, -N=CH^imine^). Elemental analysis (%): calculated for C_122_H_217_N_15_Si_13_ (2259.3 g/mol): C, 64.86; H, 9.68, N, 9.30; found: C, 64.28; H, 8.51; N, 8.62.

*G_1_-{[NCPh(o-N)]_3_[NHC(S)NH-TEMPO]} (**G_1_-PyT**, **3**)*. To a solution of G_1_-PyN (**1**) (41.8 mg, 0.045 mmol) in methanol, the radical 4-isothiocyanate-TEMPO (7.7 mg, 0,036 mmol) was added. The mixture was stirred under inert atmosphere and protected from light at room temperature for 12 h. Subsequently, the solvent was evaporated to obtain dendrimer **3** as brown oil in quantitative yield. ^1^H-NMR (CD_3_OD): δ 0.00 (s, 24H, -(C*H_3_*)_2_Si-); 0.60 (m, 24H, -SiC*H_2_*-); 1.39 (m, 10H, -SiCH_2_C*H_2_*CH_2_Si- and -SiCH_2_C*H_2_*CH_2_NH-TEMPO); 1.74 (m, 6H, (-SiCH_2_C*H_2_*CH_2_NCPh); 3.68 (m, 6H,-C*H_2_*NCPh); 7.48 (m, 3H, Ar); 7.90 (m, 3H, Ar); 8.02 (m, 3H, Ar); 8.62 (m, 3H, Ar); 8.37 (s, 3H, -N=CH^imine^). Elemental analysis (%): calculated for C_60_H_106_N_9_OSSi_5_ (1142.1 g/mol): C, 63.10; H, 9.36; N, 11.04; S, 2.81; found: C, 61.46; H, 8.59; N, 10.00; S, 2.72.

*G_2_-{[NCPh(*o*-N)]_7_[NHC(S)NH-TEMPO]} (**G_2_-PyT**, **4**).* Dendrimer G_2_-PyT was prepared through the same procedure as compound **3**, using the following reagents: G_2_-PyN (**2**) (56.0 mg, 0.025 mmol), 4-isothiocyanate-TEMPO (4.2 mg, 0.020 mmol). Dendrimer **4** was isolated as brown oil in quantitative yield. ^1^H-NMR (CDCl_3_): δ -0.05 (s, 12H, -(C*H_3_*)Si(CH_2_CH_2_CH_2_Si)_2_); -0.02 (s, 48H, -(C*H_3_*)_2_Si-); 0.61 (m, 64H, -SiC*H_2_*-); 1.42 (m, 26H, -SiCH_2_C*H_2_*CH_2_Si- and -SiCH_2_C*H_2_*CH_2_NH-TEMPO); 1.75 (m, 14H, (-SiCH_2_C*H_2_*CH_2_NCPh); 3.68 (m, 14H,-C*H_2_*NCPh); 7.46 (m, 7H, Ar); 7.89 (m, 7H, Ar); 8.00 (m, 7H, Ar); 8.61 (m, 7H, Ar); 8.35 (s, 7H, -N=CH^imine^). Elemental analysis (%): calculated for C_132_H_234_N_17_OSSi_13_ (2472.6 g/mol): C, 64.12; H, 9.54; N, 9.63; S, 1.30; found: C, 62.99; H, 8.70; N, 8.62; S, 0.82.

*G_1_-{[NCPh(*o*-N)Ru(η^6^-*p*-cymene)Cl_2_]_3_[NHC(S)NH-TEMPO]} (**G_1_-RuT**, **5**)*. To a solution of [Ru(η^6^-*p*-cymene)Cl_2_]_2_ (59.8 mg, 0.052 mmol) in methanol, G_1_-PyT (**3**) (48.1 mg, 0.16 mmol) was added dropwise at 0 °C. The mixture was stirred under inert atmosphere at room temperature for 12 h and protected from light. Subsequently, the solvent was evaporated to obtain dendrimer **5** as brown oil in quantitative yield. ^1^H-NMR (CD_3_OD): δ 0.04 (s, 24H, -(C*H_3_*)_2_Si-); 0.64 (m, 24H, -SiC*H_2_*-); 1.03 (d, 9H, -(C*H*_3_)_2_CH^cym^); 1.15 (d, 9H, -(C*H*_3_)_2_CH^cym^); 1.39 (m, 10H, -SiCH_2_C*H_2_*CH_2_Si- and -SiCH_2_C*H_2_*CH_2_NH-TEMPO); 1.82 (m, 3H, (-SiCH_2_C*H_2_*CH_2_NCPh); 2.02 (m, 3H, (-SiCH_2_C*H_2_*CH_2_NCPh); 2.29 (s, 9H, -(C*H*_3_)^cym^); 2.69 (s, 3H, -(CH_3_)_2_C*H*^cym^); 4.27 (m, 3H,-C*H_2_*NCPh); 4.69 (m, 3H,-C*H_2_*NCPh); 5.84 (m, 7H, Ar^cym^); 6.08 (m, 3H, Ar^cym^); 6.19 (m, 3H, Ar^cym^); 7.80 (m, 3H, Ar); 8.20 (m, 6H, Ar); 9.50 (s, 3H, Ar); 8.69 (s, 3H, -N=CH^imine^). Elemental analysis (%): calculated for C_90_H_148_Cl_6_N_9_ORu_3_SSi_5_ (2060.6 g/mol): C, 52.46; H, 7.24; N, 6.12; S, 1.56; found: C, 52.08; H, 7.12; N, 6.60; S, 1.62.

*G_2_-{[NCPh(*o*-N)Ru(η^6^-*p*-cymene)Cl_2_]_7_[NHC(S)NH-TEMPO]} (**G_2_-RuT**, **6**).* Dendrimer G_2_-RuT was prepared through the same procedure as for compound **5**, using the following reagents: G_2_-PyT (**4**) (29.3 mg, 0.012 mmol), [Ru(η^6^-*p*-cymene)Cl_2_]_2_ (25.7 mg, 0.084 mmol). Dendrimer **6** was isolated as brown oil in quantitative yield. ^1^H-NMR (CD_3_OD): δ -0.02 (s, 12H, -(C*H_3_*)Si(CH_2_CH_2_CH_2_Si)_2_); 0.06 (s, 48H, -(C*H_3_*)_2_Si-); 0.64 (m, 64H, -SiC*H_2_*-); 1.05 (m, 21H, -(C*H*_3_)_2_CH^cym^); 1.15 (m, 21H, -(C*H*_3_)_2_CH^cym^); 1.41 (m, 26H, -SiCH_2_C*H_2_*CH_2_Si- and -SiCH_2_C*H_2_*CH_2_NH-TEMPO); 1.88 (m, 7H, (-SiCH_2_C*H_2_*CH_2_NCPh); 2.01 (m, 7H, (-SiCH_2_C*H_2_*CH_2_NCPh); 2.29 (s, 21H, -(C*H*_3_)^cym^); 2.70 (s, 7H, -(CH_3_)_2_C*H*^cym^); 4.28 (m, 7H,-C*H_2_*NCPh); 4.69 (m, 7H,-C*H_2_*NCPh); 5.84 (m, 14H, Ar^cym^); 6.08 (m, 7H, Ar^cym^); 6.18 (m, 7H, Ar^cym^); 7.79 (s, 7H, Ar); 8.19 (m, 14H, Ar); 9.49 (s, 7H, Ar); 8.68 (s, 7H, -N=CH^imine^).Elemental analysis (%): calculated for C_202_H_332_Cl_14_N_17_ORu_7_SSi_13_ (4615.9 g/mol): C, 52.56; H, 7.25; N, 5.16; S, 0.69; found: C, 52.11; H, 7.04; N, 5.68; S, 0.65.

### 2.2. Nuclear Magnetic Resonance

Proton and carbon nuclear magnetic resonance (^1^H-NMR, ^13^C-NMR) experiments were performed on Varian Unity-500, Unity Plus-300, and Mercury Plus-300 instruments (Varian, Palo Alto, CA, USA). Deuterated chloroform (CDCl_3_) and deuterated methanol (CD_3_OD) were used as solvents. Total correlation spectroscopy (TOCSY) experiments were performed on selected compounds for further characterization insight.

### 2.3. Fourier-Transform Infrared Spectroscopy

Fourier-transform infrared (FT-IR) spectra were measured using a PerkinElmer Frontier spectrometer (Waltham, MA, USA) over KBr solid samples in the range 4000–400 cm^−1^.

### 2.4. Elemental Analysis

C, H, and N elemental analysis were performed in a microanalyzer LECO CHNS-932 (LECO, St. Joseph, MI, USA).

### 2.5. Sample Preparation for Electron Paramagnetic Resonance Analysis

#### 2.5.1. Dendrimer Stock Solution

Each dendrimer was dissolved in 1 mL of dimethyl sulfoxide DMSO:distilled water (1:9). This solution was then diluted in water to obtain a 1 mM stock solution with final 1% DMSO.

#### 2.5.2. Liposome Stock Solution

Egg lecithin was dissolved in chloroform under magnetic stirring at room temperature (r.t.) for 15 min. After chloroform evaporation, a water:phosphate buffered saline (PBS) mixture (1:1, pH = 7.2) was added to obtain a solution at a concentration of 0.05 M. The evaporated sample was added with 10 mL of distilled water in a thermostatic bath at 37 °C under stirring for 15 min. Then, 10 mL of PBS was added and stirred for 45 min. Finally, the mixture was sonicated.

#### 2.5.3. Micelle Stock Solution

Cetyltrimethylammonium bromide micelles (CTAB) were dissolved in a water/PBS buffer solution under stirring at 37 °C for 15 min.

#### 2.5.4. Final Mixtures

Final molar ratios: Dendrimer in surface groups 0.5 mM (in distilled water 0.5% DMSO), CTAB or lecithin 25 mM in buffer (PBS; pH = 7.2), and CAT12 0.125 mM in distilled water. Each sample was prepared mixing 250 µL of dendrimer stock solution and 250 µL of membrane model (CTAB/LEC) stock solutions, then stirred at 37 °C overnight and finally analyzed. We verified the low impact of the spin probe and the spin label on the system properties by changing the spin probe and the spin label concentrations and, consequently, controlling the invariability of the spectral line shape. Furthermore, the CAT12 probe was specifically synthesized and selected to mimic the surfactant structure of the CTAB and lecithin used to make the membrane models. Therefore, the eventual line shape modifications were considered informative on the system properties.

### 2.6. Electron Paramagnetic Resonance Instrumentation

Electron paramagnetic resonance spectra were recorded by means of an EMX-Bruker spectrometer operating at X band (9.5 GHz) and interfaced with a PC software WinEPR for spectra acquisition and handing, from Bruker (Bruker BioSpin, Rheinstetten, Germany). The temperature was controlled with a Bruker ST3000 variable-temperature assembly cooled with liquid nitrogen. The spectra were recorded at 37 °C ± 1. The spectra were considered valid if reproducible in at least three repeated experiments on the same sample.

### 2.7. Computation and Analysis of the Electron Paramagnetic Resonance Spectra

The computational procedure proposed by Budil et al. [33] allowed us to obtain the following main parameters of computation:

(a) The A_zz_ component of the **A** tensor for the coupling between the electron spin and the nuclear spin of the nitrogen nucleus of the nitroxide group. A_xx_ = A_yy_ = 6 G were assumed constant for simplicity. The increase in A_zz_ reflected an increase in the polarity of the nitroxide environment. For this reason, this parameter was henceforth called the polarity parameter. The error was in the second decimal and was calculated by computation; values exceeding the error produced a worse fitting between the experimental and the computed spectra, that is, we visually saw a discrepancy between the experimental line shape and the spectrum computed by using the NLSL program by Budil et al. [33]

(b) The correlation time for the rotational motion of the nitroxide group, τ, measured the microviscosity of the nitroxide environment, and, in turn, evaluated the strength of interaction occurring at the nitroxide site. This parameter was henceforth called the microviscosity parameter. The error was ± 0.001 ns for a narrow-lines spectrum and ± 0.01 ns for a broad spectrum, and was calculated by computation, that is, as indicated in (a), with values exceeding the error producing a worse fit between the experimental and the computed spectra.

In most cases, the EPR spectra were constituted by two components due to nitroxide radicals in different environments. In these cases, the experimental spectra containing the two components in different relative amounts were subtracted from each other to extract each of the components and also evaluate, by double integration of the components, their relative percentages. Then, each component was analyzed as described above.

Finally, the total intensity of well reproducible EPR spectra was evaluated by the double integral of the spectra and scaled to 100, assuming intensity = 100 for the spectrum at the highest intensity. Quantitative EPR measurements of spin concentration cannot be performed in the absence of an internal reference, but, in the present case, we trusted the intensity values only in a comparative way for a series of samples, for an indirect measure of the spin-probe solubility, and of the effect of antioxidants.

## 3. Results and Discussion

Schiff-base carbosilane dendrimers, especially those with the *N,N*-chelating 1-(2-pyridinyl)methanimine ligand, represent a promising tool for the synthesis of nanosized metallodrugs [28,29,30,31]. These dendrimers are air stable and stable in organic solvents for months; however, they are insoluble in water, methanol, and DMSO, preventing their biological evaluation through common techniques. After binding the metal complexes, they become water-soluble and biologically active. The resultant Ru(II) metallodendrimers exhibited promising antitumor activity in both in vitro and in vivo assays.

Herein, we pursue a thorough understanding about the site-site interactions between the dendrimers and the model membranes from two different points of view: the dendrimer point of view, by attaching the TEMPO spin label at the dendrimer surface; and the membrane point of view using the spin probe CAT12 that is able to enter the model membrane with the C12 chain, while the positively charged CAT group remains at the membrane surface, monitoring the interactions.

### 3.1. Synthesis and Characterization of Heterofunctional TEMPO-Labelled Ru(II) Metallodendrimers

**Scheme 1:**
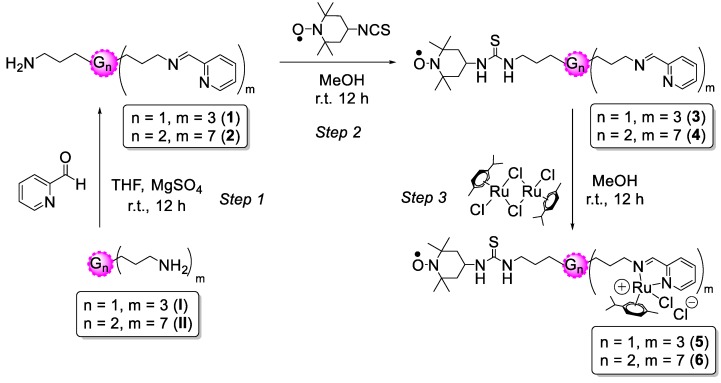
Three-step synthetic strategy to accomplish the 2,2,6,6-tetramethylpiperidine-1-oxyl (TEMPO)-labelled heterofunctional metallodendrimers G_n_-RuT (**5**,**6**).

In order to study the interactions between the dendrimers and membrane models by means of EPR, a paramagnetic species had to be introduced into the system, and a good approach was to spin label the dendritic molecule. The synthesis of the heterofunctional ruthenium metallodendrimers of carbosilane nature with the paramagnetic probe TEMPO was carried out using a three-step strategy (Scheme 1).

In the first step, we synthesized new heterofunctional dendrimers comprising multiple iminopyridine ligands, able to coordinate Ru(II) complexes, and a primary amino group, available for TEMPO binding. We decided to attach a single nitroxide group per dendrimer, regardless of the dendritic generation, for two reasons: a single radical can provide valuable information about the system, and rules out the possibility of spin–spin interactions between two proximal radicals. Furthermore, we wanted to maximize the number of iminopyridine groups and their metal complexes on the dendritic surface, and subsequently the anticancer activity. Using a statistical approach for the heterofunctionalization of the precursor dendrimers G_n_-[NH_2_]_m_ (n = 1, m = 4 (**I**), n = 2, m = 8 (**II**))[34], a condensation reaction was carried out between the amino groups present in the precursor dendrimers and 2-pyridinecarboxaldehyde, in a 1:3 ratio for the first-generation derivative, and a 1:7 ratio for the second-generation analogue, resulting in the formation of G_n_-{[NCPh(*o*-N)]_(m-1)_[NH_2_]}, (n = 1, m = 4 (G_1_-PyN, **1**); n = 2, m = 8 (G_2_-PyN, **2**)) with one free -NH_2_ group (Scheme 1) [30].

To control the partial functionalization of the precursor dendrimers, the reactions were monitored by ^1^H-NMR, adding the aldehyde portion-wise until the signal corresponding to the –CH_2_NH_2_ methylene group at 2.62 ppm integrated 25% in the case of the first-generation dendrimer and 12.5% for the second-generation compound, compared to the beginning of the reaction. The reactions were carried out under inert atmosphere using dry THF as solvent, and in the presence of MgSO_4_ as drying agent. It is important to note that the addition of the aldehyde produces a reaction color change, indicative of the formation of the new imine bond (-C=N). Once the reaction was completed, the mixture was filtered and the solvent was evaporated in vacuo obtaining G_n_-PyN dendrimers **1** and **2** as brown oils in quantitative yields. The ^1^H-NMR spectra of the Schiff-base containing heterofunctional dendritic systems confirmed the presence of a new signal from the new methinic group bound to the iminopyridine at 3.62 ppm and another new signal at 8.34 ppm corresponding to the new imine formed (Figure S1). Moreover, it was possible to observe one signal at 2.62 ppm corresponding to the methinic group attached to the amino group of the non-functionalized branch. The presence of two different types of dendritic branches was corroborated also by NMR in the first-generation dendrimer **1** spectrum because of the presence of unfolded signals, which were assigned through TOCSY-1D experiments (Figure S2). Regarding G_2_-PyN (**2**), the higher number of branches hindered the observation of unfolded signals, but it was confirmed through the –CH_2_NH_2_ signal.

In the second step, a covalent thiourea bond was formed through the reaction between the -NH_2_ group in G_n_-PyN dendrimers **1** and **2** and the isothiocyanate moiety in the TEMPO, leading to the labelled dendrimers G_n_-{[NCPh(*o*-N)]_(m-1)_[NHC(S)NHTEMPO]} (n = 1, m = 4 (G_1_-PyT, **3**); n = 2, m = 8 (G_2_-PyT, **4**)). In order to rule out the presence of non-bound TEMPO, which could hinder a clear EPR analysis, a 1:0.8 stoichiometry (dendrimer:radical) was used. This reaction was carried out under inert atmosphere, protected from light, and using methanol as solvent. After 12 h stirring at r.t., the solvent was evaporated and G_n_-PyT dendrimers **3** and **4** were obtained as brown oils in quantitative yields.

The structural characterization of compounds **3** and **4** was carried out by ^1^H-NMR using CD_3_OD as a solvent and FT-IR. The most relevant signals in the ^1^H-NMR spectrum confirmed the binding of TEMPO radical by the attenuation of the methylene group signal closest to the amino group (Figure S3). As previously reported in the literature [35], the NMR signals assigned to the TEMPO radical could not be identified because of the paramagnetic nature of this molecule. Probably for the same reason, it was not possible to identify the new signal corresponding to the methylene groups directly bound to the thiourea group. In order to compare the signal of the –CH_2_NH_2_ properly, the characterization of the ligand precursors in methanol was needed, due to the fact that these compounds were previously characterized in CDCl_3_ because of the great resolution of the signals that we observed in this solvent. With the aim of corroborating the completion of this reaction, FT-IR experiments were performed. The high-intensity broad bands belonging to the isothiocyanate groups (-N=C=S), which appeared around 2188.43 cm^−1^ in the reagent 4-isothiocyanate TEMPO, disappeared once attached to our dendrimers (Figure S5).

In the final step, the ruthenium precursor [Ru(η^6^-*p*-cymene)Cl_2_]_2_ was coordinated to the *N,N*-chelate ligands present at the periphery of the dendrimers. G_n_-PyN dendrimers **3** or **4** were slowly added to a methanol solution of the Ru(II) precursor at 0 °C. The reaction mixture was stirred for 12 h and then evaporated in vacuo, obtaining, in quantitative yields, the labelled metallodendrimers G_n_-{[NCPh(*o*-N)]_(m-1)_[NHC(S)NHTEMPO]} (n = 1, m = 4 (G_1_-RuT, **5**); n = 2, m = 8 (G_2_-RuT, **6**)) as water-soluble brown solids (Scheme 1).

The ^1^H-NMR spectra of final Ru(II) complexes, G_1_-RuT and G_2_-RuT (indicated as **5** and **6** in Scheme 1), revealed the formation of two new Ru–N bonds by the shift towards higher frequencies of the signal assigned to the methylene group closer to the imine nitrogen, the proton of the imine itself, as well as of the methinic groups present in the pyridine ring (Figure S4). The addition of the Ru(II) precursor was completely controlled by NMR in order to avoid having an excess of the metal precursor.

### 3.2. Electron Paramagnetic Resonance Study of TEMPO-Labelled Dendrimers in the Absence and Presence of Model Membranes

The spin-labelling technique is a very useful tool that offers an in situ reporter at the dendrimer surface about the eventually occurring interactions. An accurate computer-aided analysis of the EPR spectra allowed us to obtain specific information about the type and strength of interactions and the structural variations at the G_n_-PyT and G_n_-RuT dendrimer/model membrane interface.

**Figure 2:**
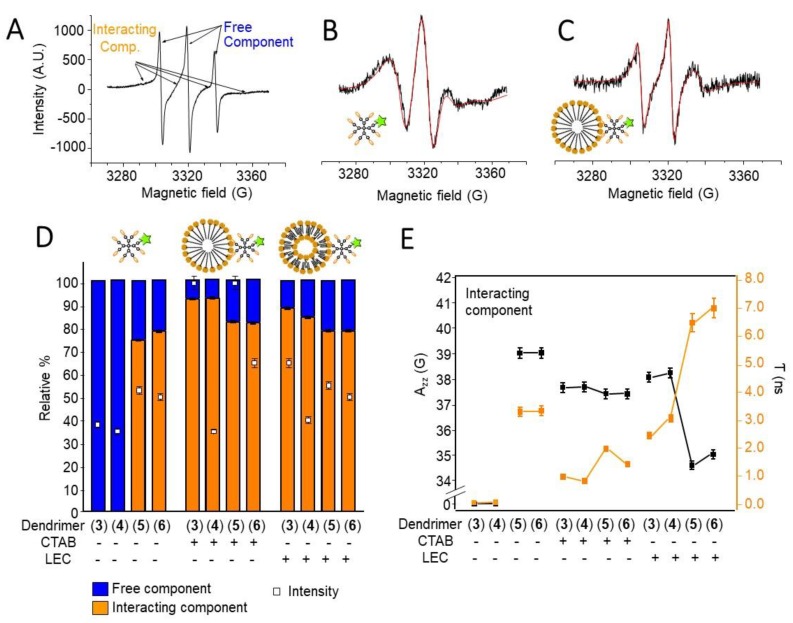
Experimental spectra and computation spectra/parameters for the EPR spectra of the labelled dendrimers G_n_-PyT and G_n_-RuT **3–6** in the absence and presence of the model membranes (cetyltrimethylammonium bromide micelles (CTAB) and lecithin liposomes (LEC)). (**A**) Experimental EPR spectrum of G_1_-RuT (**5**) in Phosphate Buffered Saline , selected as an example. Arrows indicate the main features of the two components constituting the spectra (termed Free and Interacting); (**B,C**) experimental and computed Interacting components of G_1_-RuT (**5**) alone or in the presence of CTAB, obtained after subtraction of the Free component. The spectra are normalized in height. (**D**) Total intensity of the EPR spectra (squares, assuming I = 100% for the spectrum of the labelled dendrimers in the absence of model membranes) and relative percentage of Interacting component (bars). (**E**) Polarity parameter, A_zz_, and microviscosity parameter, τ, for the Interacting component.

Figure 2A shows, as an example, the EPR spectrum of G_1_-RuT (**5**) in PBS buffer. The arrows in the figure indicate the main features of the two components constituting the spectra. The first component is composed of three narrow lines, which are characteristic of fast moving nitroxide radicals, and it is henceforth called the Free component. The second broader component shows a partial resolution of the anisotropies, evidenced by the shift of the main peaks. The resolution of the anisotropies arose in the case of slow motion of the nitroxide group, due to an increase of the local microviscosity, which, in turn, was related to interactions occurring at the nitroxide environment. For these reasons, this component is henceforth called Interacting component. The occurrence of both these components for the labelled dendrimers in the absence of membrane models indicated that the free and interacting labels were already present at the surface of the labelled dendrimers, due to a different location of the labels themselves, one more external and the other more internal at the dendrimer interface; however, as we will discuss later, both components were affected by the interactions with the model membranes.

The occurrence of both components allowed us to perform subtractions between spectra to extract each of the components and also evaluate, by double integration of the components, their relative percentages. The components were then also computed to extract parameters providing structural and dynamical information (Table 1, see the experimental section for details). Figure 2B,C show as examples the experimental and computed Free and Interacting components for G_1_-RuT (**5**) alone and in the presence of CTAB. Further examples of experimental and computed spectra of labelled dendrimers are shown in Figure S6.

TEMPO-labelled dendrimers G_n_-PyT (**3** and **4**, entries 1 and 2 in Table 1, Figure 2D) only showed a Free component, since the radical group was free to move at the dendrimer surface. The computation revealed a very polar environment and a high mobility (low microviscosity), almost equivalent to that found for TEMPO radicals in water. The minor differences in A_zz_ and τ between these two dendrimers can be ascribed to the larger size of G_2_-PyT, which produced a small increase in microviscosity. The addition of CTAB micelles to these dendrimers (entries 3 and 4 in Table 1) altered the Free component, inducing a decrease of the polarity and mobility (increase in the microviscosity parameter τ) in agreement with a partial insertion of the nitroxide label into the micellar structure. Unexpectedly, the addition of liposomes provoked a lower change of the Free component (entries 5 and 6 in Table 1), indicating that the fast moving labels interact weakly with the liposome structure and mainly remained confined at the interface.

The attachment of Ru(II) complexes to the labelled dendrimers revealed a different interacting behavior. In TEMPO-labelled metallodendrimers G_n_-RuT (**5** and **6**, entries 7 and 8 in Table 1, Figure 2D), the Free component already showed, in the absence of model membranes, a slightly lower polarity and a higher microviscosity if compared to G_n_-PyT. The addition of the micelles and the liposomes poorly modified these values (Entries 9–12 in Table 1). Therefore, for the Ru-containing dendrimers, the external free labels were already partially hindered in their rotational mobility at the dendrimer surface, and the interactions with the model membranes provided only small changes in the microviscosity and polarity of the radical environment.

A different but complementary story was told by the computation parameters (the polarity parameter, A_zz_, and the microviscosity parameter, τ) of the Interacting component (Table 1 and Figure 2E). It is useful to discuss these parameters together with other parameters, i.e., (a) the total intensity, evaluated by the double integral of the spectra and scaled to 100 (assuming I = 100% for the labelled dendrimers in the absence of model membranes), reported in Figure 2D, squares; and (b) the relative percentage of the Interacting component, reported in Figure 2D, bars.

The total intensity of the EPR spectra measured the dendrimer solubility. In the absence of model membranes, the intensities for G_n_-PyT were quite low, and slightly increased for G_n_-RuT. However, the intensity increased in the presence of CTAB, revealing a significant improvement of solubility due to the interactions with micelles, mainly for first-generation dendrimers. Conversely, the presence of LEC showed no significant changes on the dendrimers’ solubility, except for G_1_-PyT. The relative percentage of Interacting component (Figure 2D, bars) quantifies the interacting capacity of the dendrimers. As previously mentioned, the Interacting component was absent for G_n_-PyT when the model membranes are not present. Conversely, it appeared at a quite high relative extent in the presence of model membranes, mainly for CTAB. The Ru-containing dendrimers, G_n_-RuT **5** and **6**, showed an Interacting component already in the absence of model membranes, due to the interaction of the TEMPO label with the Ru complex. This percentage slightly increased by adding model membranes, especially CTAB micelles, monitoring the dendrimer/membrane interactions.

As shown in Figure 2E, the environmental polarity measured by A_zz_ decreased from the Free to the Interacting component, and from the absence to the presence of model membranes, indicating that the label responsible of the Interacting component was partially inserted into the lipid core of the aggregate. This effect was quite significant for G_n_-RuT with LEC, while the G_n_-PyT dendrimers showed a better insertion into CTAB with respect to LEC. A perfect agreement was observed for the microviscosity parameter τ, giving the highest values for the Ru-metallodendrimers in the presence of LEC. Conversely, the CTAB micelles provided a more fluid environment with respect to the liposomes for the dendrimer surface groups partially entering the lipid structure, confirming a better interaction with the micelles.

### 3.3. Electron Paramagnetic Resonance Study of Unlabelled Dendrimers Using CAT12 Probe in the Absence and Presence of Model Membranes: Comparison with TEMPO-Labelled Dendrimers

In order to gain further insight into the interacting abilities of the dendrimers, we selected the family of homofunctional dendrimers comprising iminopyridine groups in the periphery G_n_-Py (first- and second-generation dendrimers 1* and 2*, Figure 1, as well as the mononuclear ligand 0*) [30]. Considering their diamagnetic properties, the use of a spin probe was required to analyze by EPR the interactions occurring between the dendrimers and the model membranes. The CAT12 probe was selected to provide complementary information with respect to those obtained by using labelled dendrimers. CAT12, being a surfactant, was expected to enter the model membrane with the carbon chain, while the positively charged CAT group localized at the membrane surface. This location allowed us to obtain information about the interactions between the dendrimer and the model membrane from the membrane side, while the labels at the dendrimer surface obviously provided information from the other side, the dendrimer surface.

The selection of the CAT12 probe was also based on the similarity between the EPR spectra obtained from the labelled dendrimers and CAT12 in the presence of both the dendrimer and the model membrane. The same line shape was obtained by using the homofunctional G_n_-Py family and the heterofunctional G_n_-PyN family, and, as shown in Figure 2A, the spectra were again constituted by the two components, Free and Interacting. In both cases, labelled and unlabelled dendrimers, the subtraction procedure between spectra allowed us to extract the two components, evaluate their relative percentages, and compute both of them to obtain the mobility and polarity parameters, which provided information about the dendrimer–model membrane interactions, as described above for the TEMPO-labelled dendrimers. Similarly, the same computation parameters were analyzed for the CAT12 probe as for the labelled dendrimers. In this case, we also found spectral variations over the equilibration time, and, therefore, results are shown and discussed for spectra recorded both after a short time (15 min) and a longer time (24 h) of mixture equilibration. The equilibration time impact was similar for the homofunctional G_n_-Py and the heterofunctional G_n_-PyN dendrimers. Then, the equilibration time effect will only be discussed for the homofunctional G_n_-Py dendrimers, while, for simplicity, the results for the heterofunctional G_n_-PyN dendrimers are only shown for the 15 min spectra to underline the differences between the two series of dendrimers. Further examples of experimental and computed spectra of non-labelled dendrimers in the absence and presence of model membranes are shown in Figure S7.

#### 3.3.1. CAT12 Electron Paramagnetic Resonance Study of the Interactions between Homofunctional G_n_-Py Dendrimers and Model Membranes

**Figure 3:**
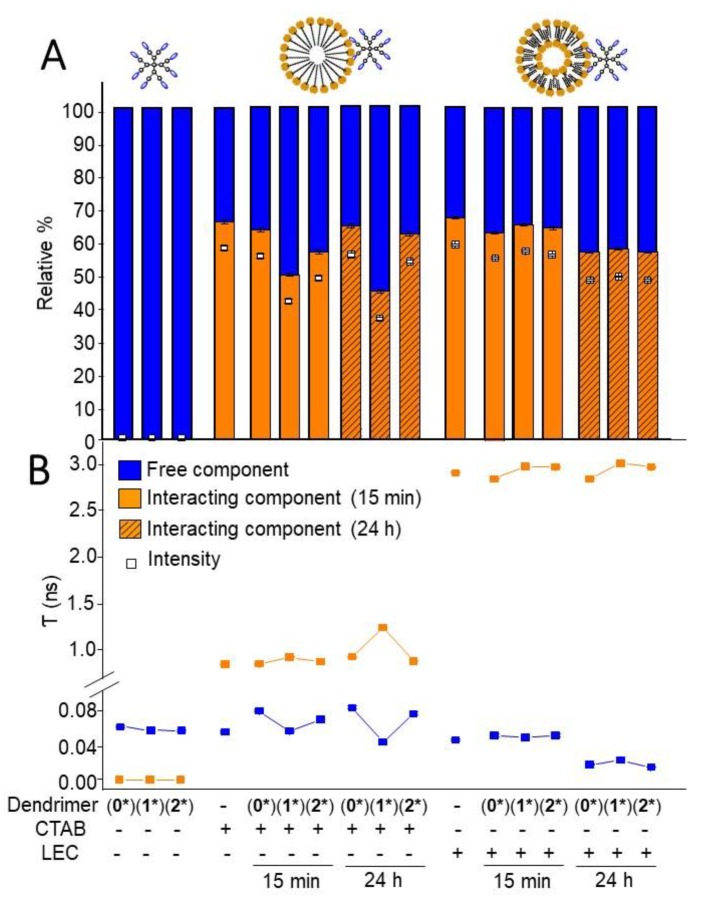
Parameters obtained from the analysis of CAT12 EPR spectra of the unlabeled dendrimers G_n_-Py in the absence and presence of the model membranes (CTAB micelles and LEC liposomes). (**A**) Relative percentages of interacting component (bars) for CTAB and LEC samples in the absence and presence of G_n_-Py dendrimers at 15 min and 24 h equilibration times, and intensity values (squares), in percentages, assuming 100% as the highest intensity. (**B**) Microviscosity parameter, τ, for both the Free and Interacting components.

The relative intensity and relative percentage of interacting component of CAT12 in CTAB and LEC samples in the absence and presence of the G_n_-Py dendrimers are shown in Figure 3A. The CAT12 probe is quite soluble in the PBS solution (intensity about 85%), but in the absence of model membranes the intensity decreased by adding G_0_-Py and G_2_-Py and increased by adding G_1_-Py, which was assumed as 100% of intensity. This behavior helped to clarify some properties of the different generation dendrimers in respect to their interacting ability, since CAT12 was interacting by means of both polar and hydrophobic interactions with the dendrimers, as already described in previous studies, and therefore followed the fate of the dendrimers [36]. It can be concluded that G_1_-Py showed the highest solubility and interacting ability with the medium, while G_0_-Py had the lowest solubility, probably due to the higher hydrophobicity. The opposite experiment, using a solution of model membranes in the absence of the dendrimers, revealed a decrease in the intensity with respect to the CAT12 probe in PBS. It can be explained by the entry and high concentration of the probes at the interface of the aggregates provoking strong spin–spin interactions, which led to very broad lines, no more visible in the spectra. The decrease in intensity was higher for LEC with respect to CTAB, probably due to the high packing of the LEC structure.

In the presence of both the dendrimer and the model membrane, the trend found by changing generation was also maintained in the presence of CTAB and LEC and at the different equilibration times. For CTAB dendrimer samples, the intensity was in between the ones for the dendrimer alone and for the CTAB alone in the case of G_1_-Py and G_2_-Py, while for G_0_-Py, the two components’ mixture further decreased the solubility. For LEC dendrimer samples, the intensity decreased (~30% maximum) as the radicals were concentrated at the dendrimer/liposome interface. The significant decrease in intensity from 15 min to 24 h in the presence of LEC indicated that the radicals progressively approached each other at the aggregate/dendrimer interface, while the opposite happened in CTAB micelles, where the radicals redistributed better over time into the micelles because of their fluidity when compared to the highly packed liposomes.

Figure 3A also shows the relative percentages of interacting components for CTAB and LEC samples in the presence of the various dendrimers at 15 min and 24 h equilibration times. While the dendrimers alone only showed a Free component, the probe in the model membranes in the absence and presence of the dendrimers showed both the Free and the Interacting components. For CTAB samples, the variation in relative percentage of Interacting component followed an opposite trend with respect to the intensity variation, being the lowest for G_1_-Py and the highest for G_0_-Py. The high solubility of CAT12 with G_1_-Py dendrimer well justified the low interacting component percentage, as this dendrimer, when interacting with the micelles, extracted the probes from the CTAB interface and promoted their solubilization, with this effect continuing over time. The percentage of Interacting component was the highest for G_0_-Py, almost comparable to the percentage for CTAB alone,. G_1_-Py showed an intermediate situation, however, for G_0_-Py and furthermore for G_2_-Py, the percentage of interacting component in the presence of CTAB increased over time, suggesting a progressive increase of interactions over time. 

Similarly to CTAB, the relative percentage of interacting probes for LEC decreased from the absence to the presence of the dendrimers; however, in this case, G_1_-Py dendrimer showed a higher percentage with respect to the other generations. The LEC liposomes were well packed and the dendrimer–LEC interactions were poorly able to modify the liposome structure. However, over time, the percentage of interacting probes further decreased in line with the decrease in intensity, indicating an increased dendrimer/liposome interaction, which provided an increased local concentration of probes at the interface with consequent strong spin–spin interactions and the disappearance of these probes from contributing to the EPR spectrum.

Further information comes from the parameters A_zz_ and τ obtained by spectral computation (Figure 3E and Figure S8). First of all, we noted that the Free component showed almost equivalent τ and A_zz_ values for CTAB in the absence of the dendrimers and for the dendrimers in the absence of CTAB. This meant that the CAT12 heads were at the external surface of the micelles and the dendrimers, feeling the same environment (the water solution). The polarity for the Free probes almost did not change in function of generation and equilibration time. Conversely, the microviscosity for the Free probes increased for CTAB samples in the presence of G_0_-Py and G_2_-Py, while it decreased in the presence of G_1_-Py samples, mainly at 24 h. Since for G_1_-Py + CTAB samples the intensity was higher than for the other generations and the relative percentage of interacting probes decreased, it was clear that the interaction between G_1_-Py dendrimer and the micelles provoked the extraction of probes from the micelles to the solution, thus increasing both the relative quantity of Free probes and their freedom of motion. This behavior may be accounted for by a modification of the micellar structure due to interactions with G_1_-Py. The fact that the microviscosity of the Interacting component for G_1_-Py + CTAB increased, mainly after 24 h, and that the polarity decreased, further demonstrated the structural variations of the micelles when interacting with G_1_-Py. A higher packing of the surfactants accounted well for the extrusion of some probes from the micelles while the probes remaining inside the micelles more strongly interacted with the dendrimer surface, also partially approaching the less polar region. A quite different behavior was found for G_0_-Py and G_2_-Py—for them, the relative percentage of Interacting component changed poorly with respect to the micelles in the absence of the dendrimers, and the Free probes increased their microviscosity since they remained trapped at the dendrimer–micelle interphase. The interactions were weaker with G_0_-Py and G_2_-Py if compared to G_1_-Py because of an unfavorable balance between polar and hydrophobic forces, and the interacting probes showed a smaller decrease in polarity, more significant for G_0_-Py than for G_2_-Py.

For LEC liposomes, the differences as a function of generation were much lower. The LEC structure was preserved after 15 min, but after 24 h all the dendrimers provoked a decrease of both the percentage of interacting probes and the microviscosity of free probes, indicating a structural variation connected with the extraction of probes from the liposomes to the solution due to the dendrimer/liposome interactions. The interaction strength of the CAT group at the liposome interface with G_0_-Py was quite low, while it increased with both G_1_-Py and G_2_-Py because of the higher density of polar surface groups.

#### 3.3.2. Electron Paramagnetic Resonance Study of the Interactions between Heterofunctional G_n_-PyN Dendrimers and Model Membranes Using CAT12 as Spin Probe

Once we had established the interactions between homofunctional iminopyridine dendrimers G_n_-Py and the model membranes, we addressed the influence of leaving one –NH_2_ group unmodified (Figure 1, compounds **1** and **2**). Interesting variations of the EPR parameters were observed for the heterofunctional dendrimers G_n_-PyN, compared to the homofunctional counterparts G_n_-Py.

**Figure 4:**
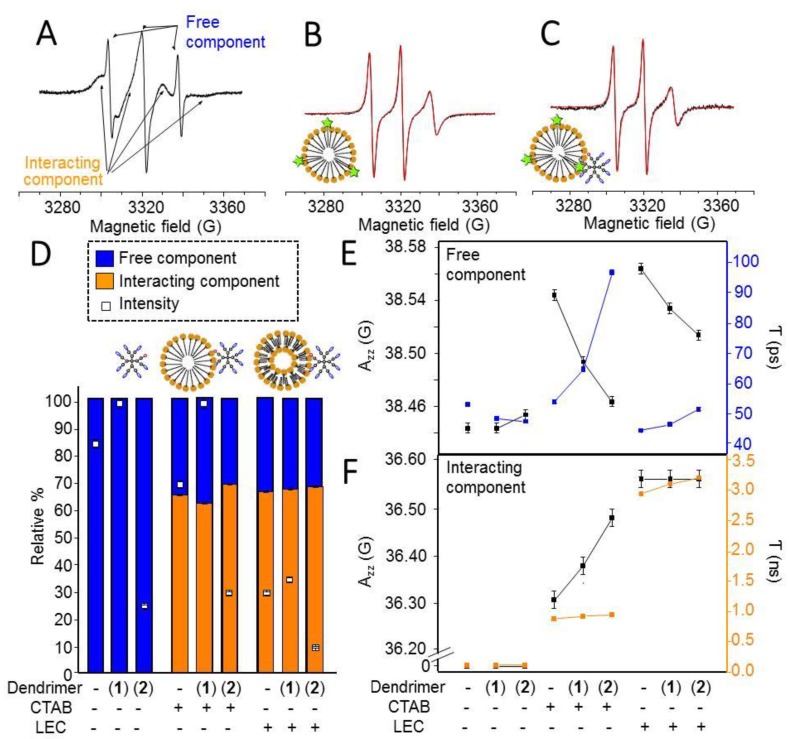
Experimental spectra and computation spectra/parameters for the CAT12 EPR spectra of the unlabeled heterofunctional dendrimers G_n_-PyN in the absence and presence of the model membranes (CTAB micelles and LEC liposomes). (**A**) EPR spectrum of CAT12 for G_1_-PyN (**1**) in the presence of LEC; (**B, C**) experimental (black) and computed (red) spectra of the interacting component for CAT12 in CTAB sample, alone and in the presence of G_2_-PyN (**2**); (**D**) Relative percentage of Interacting component (bars), and total intensity (squares) as a percentage, where 100% intensity was ascribed to the spectrum at the highest intensity; (**E, F**) A_zz_ values (left axis) and τ values (right axis) for the Free and the Interacting components. PBS sample was evaluated in the absence of dendrimers and model membranes.

For a matter of clarity, an example of EPR spectrum obtained for CAT12 in G_1_-PyN+LEC is shown in Figure 4A, again constituted by the two components, Free and Interacting. Examples of computations of the interacting components are shown in Figure 4B,C, while the main parameters obtained from the analysis of the EPR spectra are shown in Figure 4D,E.

As already found for G_n_-Py dendrimers, the intensity (Figure 4D, squares) measuring the probe solubility, was higher for the first-generation dendrimer when compared to the other generations. The solubility of CAT12 probes increased in the presence of G_1_-PyN because of a good balance between polar and hydrophobic interactions. This effect was maintained for G_1_-PyN in the presence of CTAB micelles. However, the absence of G_1_-PyN (CTAB alone) decreased the intensity, probably because some probes concentrated into the micelles provoking strong spin–spin interactions, which led to significant line broadening and the spectral contribution being no longer visible in the spectra. A similar effect may justify the significant decrease in intensity found for the liposomes in the absence of dendrimers, as already discussed in the previous section. For G_2_-PyN, the CAT12 solubility was low both in the absence and in the presence of CTAB, and reached a very low value (below 10%) in the presence of LEC. The interactions between G_2_-PyN and LEC provoked a further local increase in probe concentration, increasing spin–spin interactions.

After CAT12 aggregation into the micelles, which led to the intensity decrease in the presence of G_2_-PyN, the remaining non-aggregated probes showed an increase in the relative percentage of interacting component due to G_2_-PyN-micelle interactions (Figure 4D, bars). This effect, not observed for G_2_-Py, was undoubtedly due to the presence of the -NH_2_ group. Conversely, G_1_-PyN showed a decrease in the percentage of the interacting component, even if not as low as for G_1_-Py. Therefore, also in this case, G_1_-PyN interactions with CTAB produced an extraction of probes from the micelles to the solution, related to a restructuration of the micelles upon interaction with the dendrimer. However, the presence of the -NH_2_ group, instead of another iminopyridine ligand, decreased this effect, and promoted the dendrimer–micelle interactions preserving the micellar structure.

For LEC liposomes, the presence of the -NH_2_ group inverted the trend for the variation of the percentage of interacting component. Indeed, for G_n_-Py dendrimers, the percentage diminished in the series LEC > LEC+G_1_-Py > LEC+G_2_-Py, while for G_n_-PyN dendrimers the interacting percentage slightly increased in the series LEC < LEC+G_1_-PyN < LEC+G_2_-PyN. The interactions were preferentially at the polar surface of the liposomes, as also demonstrated by the polarity parameter A_zz_ (Figure 4F).

For the polarity and microviscosity parameters, the Free component was also informative (Figure 4E). This Free component arose from probes in solution, but was affected by the surface of dendrimers and model membranes. By adding the dendrimers to CTAB micelles, the polarity decreased because of the approaching of the less polar dendrimer to the model membrane. This approaching partly reduced the mobility (increasing the microviscosity). For LEC, by adding the dendrimers, the freedom of motion and the polarity were reduced because of dendrimer/membrane interactions, and this effect was stronger by increasing generation.

In respect to the Interacting component (Figure 4F), both polarity and microviscosity increased for the model membranes from the absence to the presence of the dendrimers, by increasing generation, and from CTAB to LEC. Also in this case, some trends were inverted with respect to G_n_-Py dendrimers. For instance, the microviscosity, measuring the strength of interaction, decreased from G_1_-Py to G_2_-Py, while it increased from G_1_-PyN to G_2_-PyN; in detail, in respect to CTAB, G_1_-Py was more interactive than G_1_-PyN, while G_2_-Py was less interactive than G_2_-PyN. For the polarity, G_1_-Py showed lower polarity than G_1_-PyN, while G_2_-Py showed higher polarity than G_2_-PyN. These results revealed a different interaction mechanism between the different dendrimers and CTAB micelles, being G_1_-Py able to partially enter the micelles, approaching less polar sites and providing a stronger interaction with the micelles. The presence of -NH_2_ at the G_1_-PyN surface hindered the penetration into the micelle, favoring interactions at the charged external surface. For G_2_-Py, the higher density of surface groups, the lower availability of the low polar dendrimer core, and the higher structural rigidity impeded the penetration into the micelles. Therefore, the interaction was only at the external surface, and the presence of -NH_2_ at the G_2_-PyN surface favored polar and electrostatic interactions with the micelles.

The higher A_zz_ and τ parameters for LEC with respect to CTAB were due to the more dense and viscous liposome structure at the interface. The increased microviscosity and polarity parameters in the presence of the dendrimers were clearly reporting the occurrence of electrostatic interactions between the dendrimers and the liposome surface, which became stronger by increasing generation due to the higher density of surface groups for the higher generation. If compared to G_n_-Py, G_n_-PyN dendrimers showed higher strength of interaction and higher polarity. Furthermore, G_2_-PyN showed stronger interactions with respect to G_1_-PyN, while the opposite held for G_n_-Py dendrimers. This confirmed that the dendrimer–liposome interactions were mainly polar/electrostatic between the polar/charged surfaces. Therefore, the -NH_2_ group favored these kinds of interactions.

The last interesting finding was the agreement between the results obtained for the labelled dendrimers (**3**–**6**) and for the unlabeled ones (**1**–**2**, 1*–2*). Both points of view suggested the partial insertion of the dendrimer surface groups into the surfactant aggregates, mainly CTAB micelles, and the occurrence of both polar and hydrophobic interactions, while dendrimer–LEC interactions involved more polar interactions between surface groups. Finally, the G_1_-Py interactions were significantly favored, mainly with CTAB, but both the presence of -NH_2_ and TEMPO moieties perturbed these interactions, partially preventing the entry of dendrimer surface groups into the micellar core.

## 4. Conclusions

A selective and efficient anticancer therapy requires an efficient uptake of the drug by cancer cells, which in turn depends on the ability of the drug to interact and penetrate the cell membrane. Using model cell membranes (CTAB and LEC), we evaluated in situ the interactions performed by a new family of differently decorated carbosilane dendrimers through a computer-assisted EPR analysis. The analysis of the EPR spectra provided parameters informative of the interacting ability of the dendrimers, and the type and strength of interactions in function of the effect of a metal (Ru(II)), the type of model membrane, a partial functionalization, and generation.

In order to report the interactions from the dendrimer point of view, we synthesized new heterofunctional Ru(II) metallodendrimers labelled with a TEMPO radical. The new nanotheranostic G_n_-RuT dendrimers can potentially exhibit an antitumor effect while monitoring in real time drug release and distribution. In the synthetic route, interesting precursors were prepared—The G_n_-PyN family, with multiple iminopyridine ligands and a single –NH_2_ group, and the G_n_-PyT family, with multiple iminopyridine ligands and a TEMPO label. These subtle changes of a single group later revealed important differences regarding the interactions between the dendrimers and the CTAB and LEC models. Initially, a comparative EPR study was performed on the labelled dendrimers G_n_-PyT and G_n_-RuT in the absence and presence of the model membranes. It was found that the G_n_-PyT dendrimers partially penetrated the micelle structure, with G_1_-PyT being more interactive than G_2_-PyT. In the liposomes the interactions occurred at the external surface, with G_2_-PyT being more interactive than G_1_-PyT. In the metallodendrimers, the TEMPO group interacted with Ru(II) ions but gained freedom entering the micellar structure; otherwise their mobility was slowed down by interacting with the LEC surface. Therefore, for both G_n_-PyT and G_n_-RuT, the interactions were perturbative of the CTAB micellar structure, but preserve the LEC structure, mainly occurring at the external interface.

The EPR results from the spin-labelled dendrimers were compared and integrated with those obtained by both the unlabeled dendrimer precursors (G_n_-PyN) and the homofunctional iminopyridine dendrimers (G_n_-Py). In these last cases, the CAT12 probe demonstrated the provision of complementary information with respect to those obtained with the labelled dendrimers. Also in this case, the computer aided EPR analysis allowed us to extract similar parameters as those obtained by means of the TEMPO label, but the point of view provided by CAT12 was on the model membrane side, since this spin probe is deeply inserted into the surfactant aggregates. It was found, similar to G_n_-PyT, that G_1_-Py interacts better with CTAB, while G_2_-Py interacts better with LEC. G_1_-Py and G_1_-PyN interactions with CTAB produced an extraction of probes from the micelles to the solution, which was related to a restructuration of the micelles in agreement with the results from the labelled dendrimers. The presence of the NH_2_ group at the G_2_-PyN surface promoted polar and electrostatic interactions with the micelles instead of penetration of dendrimer branches into the micellar structure. Therefore, the presence of NH_2_ at the G_1_-PyN dendrimer surface impeded the penetration into the micelle, favoring interactions at the charged external surface.

Clearly the EPR analysis demonstrated itself to be very useful in providing detailed information on the mechanism of interactions of the dendrimer with a model cell membrane, in view of the use of these new dendrimers as anticancer drugs. A second manuscript containing comparative EPR experiments and biological tests using both healthy and tumor cells in the absence and presence of the dendrimers is currently in preparation.

## Figures and Tables

**Table 1:** Main parameters of computation for the labelled dendrimers G_n_-PyT and G_n_-RuT in the absence and presence of model membranes.

Entry	Sample	Components	A_zz_ (G)	τ (ns)
1	G_1_-PyT (**3**)	Free (single)	39.21/-	0.014/-
2	G_2_-PyT (**4**)	Free (single)	39.12/-	0.017/-
3	G_1_-PyT (**3**) + CTAB	Free + Interacting	38.72/37.68	0.21/0.87
4	G_2_-PyT (**4**) + CTAB	Free + Interacting	38.68/37.70	0.25/0.73
5	G_1_-PyT (**3**) + LEC	Free + Interacting	39.01/38.07	0.050/2.35
6	G_2_-PyT (**4**) + LEC	Free + Interacting	38.98/38.20	0.055/3.05
7	G_1_-RuT (**5**)	Free + Interacting	38.82/39.00	0.22/3.31
8	G_2_-RuT (**6**)	Free + Interacting	38.85/39.00	0.22/3.35
9	G_1_-RuT (**5**) + CTAB	Free + Interacting	38.82/37.40	0.22/1.90
10	G_2_-RuT (**6**) + CTAB	Free + Interacting	38.85/37.41	0.22/1.38
11	G_1_-RuT (**5**) + LEC	Free + Interacting	38.82/34.60	0.22/6.45
12	G_2_-RuT (**6**) + LEC	Free + Interacting	38.85/35.00	0.22/7.00

## Supplementary Materials

The following are available online at https://www.mdpi.com/2218-273X/9/10/540/s1, Figure S1: ^1^H- and ^13^C{^1^H}-NMR spectra of compound **1**. Figure S2: TOCSY 1D spectrum of compound **1**. Figure S3: ^1^H-NMR spectrum of compound **3**. Figure S4: ^1^H-NMR spectrum of compound **5**. Figure S5: FT-IR spectra of precursor 4-isothiocyanateTEMPO and compound **3**. Figure S6: Examples of experimental and computed spectra of labelled dendrimers in the absence and presence of model membranes. Figure S7: Examples of experimental and computed spectra of non-labelled dendrimers in the absence and presence of model membranes. Figure S8: A_zz_ values obtained by computing the Free and the Interacting components for CTAB for homofunctional dendrimers Gn-Py.
