# Supplementary material for: Exploring the Interactions of Ruthenium (II) Carbosilane Metallodendrimers and Precursors with Model Cell Membranes through a Dual Spin-Label Spin-Probe Technique Using EPR
Source: Biomolecules. 2019 Sep 27;9(10):540. doi: 10.3390/biom9100540 (PMC6843795; doi:10.3390/biom9100540)
Supplement: Supplementary file 1 [file biomolecules-09-00540-s001.pdf]

Electronic Supporting Information

Exploring the interactions of ruthenium(II) carbosilane metallodendrimers and precursors with model cell membranes through a dual Spin-Label Spin-Probe technique using EPR

Riccardo Carloni[1†,] Natalia Sanz del Olmo[234†], Paula Ortega[234], Alberto Fattori[1], Rafael Gómez[234], Maria Francesca Ottaviani[1], Sandra García-Gallego [234], Michela Cangiotti[1] and F. Javier de la Mata[234]**

[a]Department of Pure and Applied Sciences, University of Urbino “Carlo Bo”, Urbino, Italy.

[b]Department of Organic and Inorganic Chemistry, and Research Institute in Chemistry Andrés M. del Río” (IQAR), University of Alcalá, Madrid, Spain;

[c]Networking Research Center on Bioengineering, Biomaterials and Nanomedicine (CIBER-BBN), Spain.

[d]Institute Ramón y Cajal for Health Research (IRYCIS), Spain.

Figures

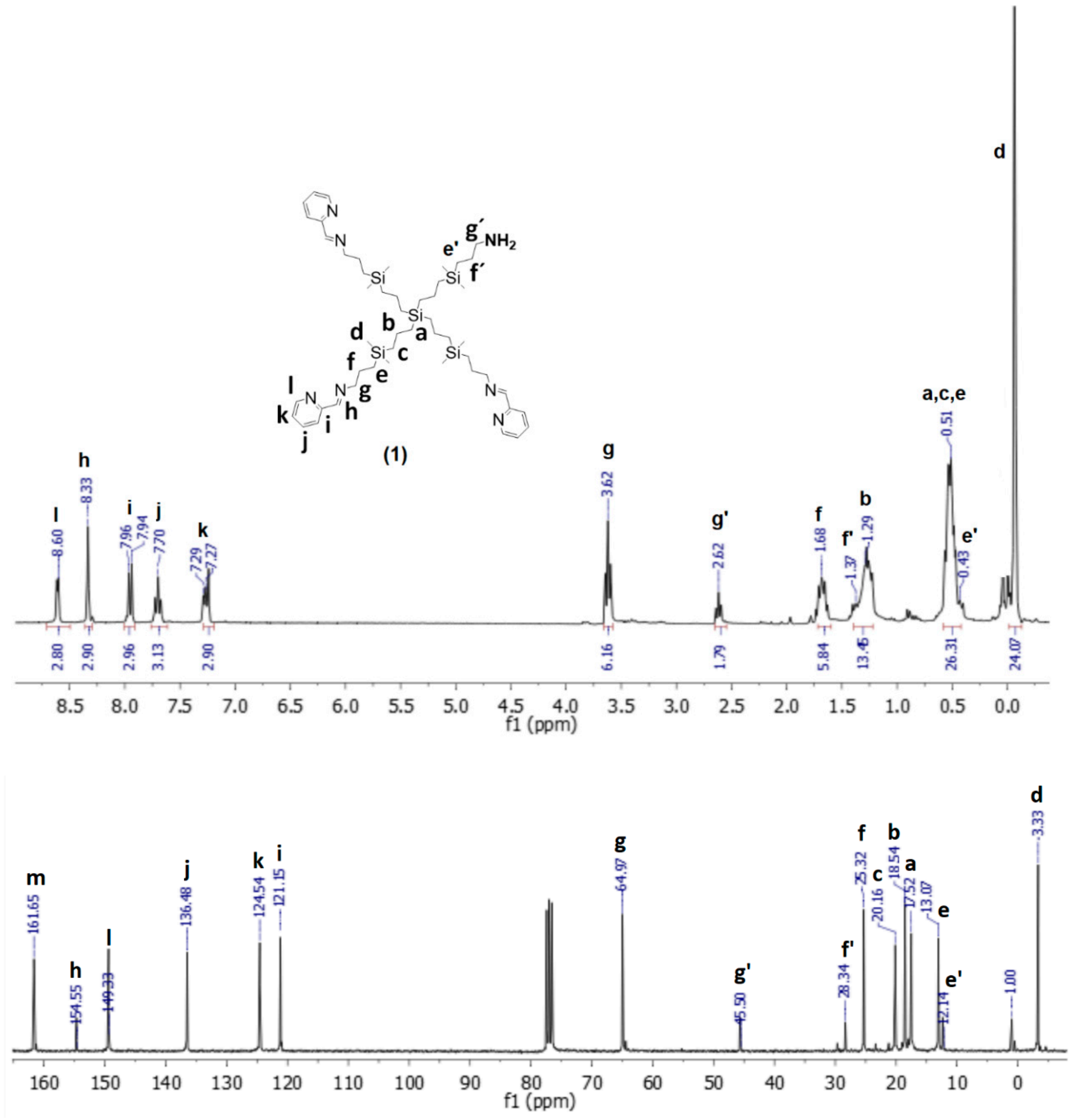

Figure S1. ^{1}H- and ^{13}C{^{1}H}-NMR spectra of compound **1** in $CDCl_3$.

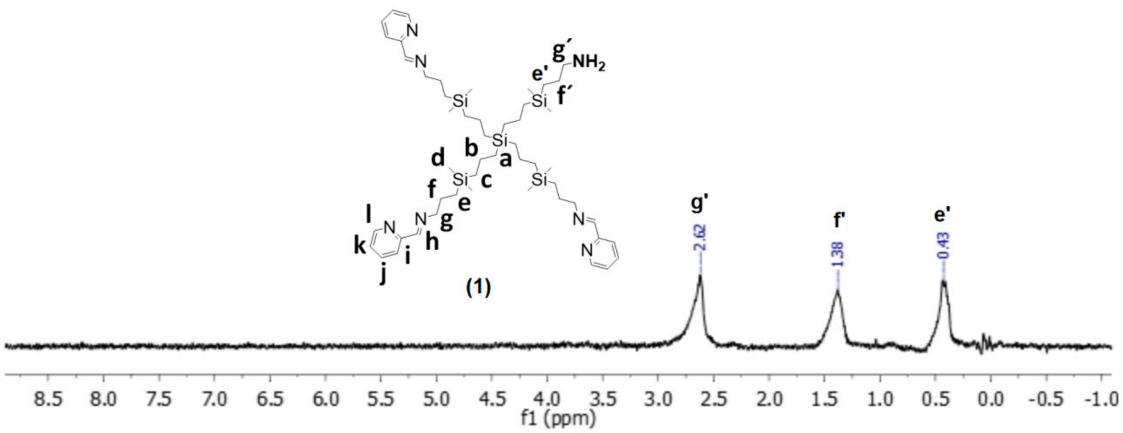

Figure S2. TOCSY 1D spectrum of compound **1** in $CDCl_3$.

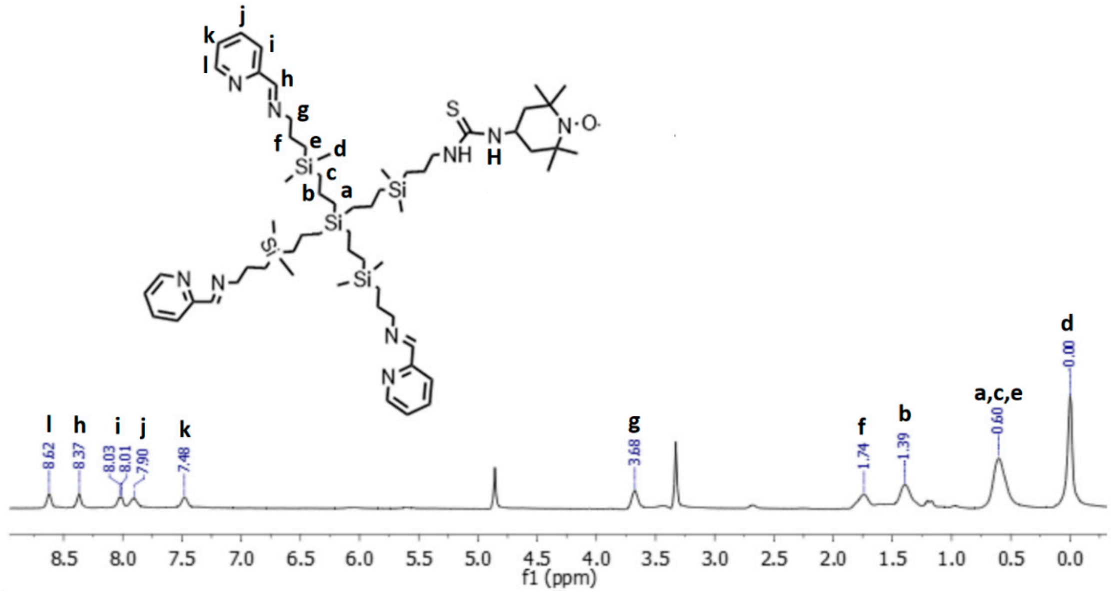

Figure S3. ^{1}H-NMR spectrum of compound **3** in CD_3OD.

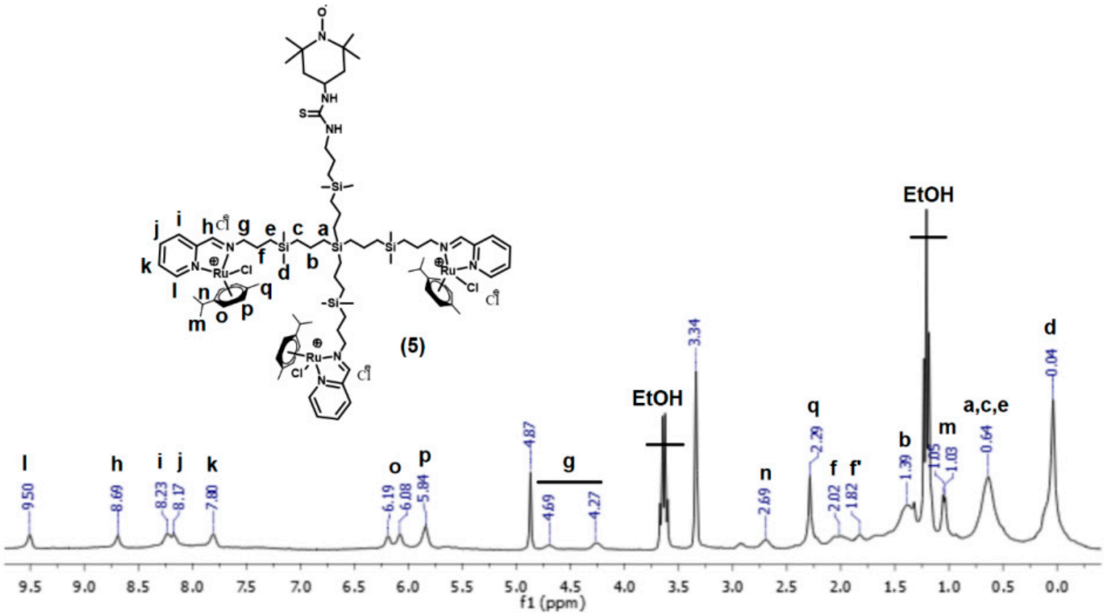

Figure S4. ^{1}H-NMR spectrum of compound **5** in CD_3OD.

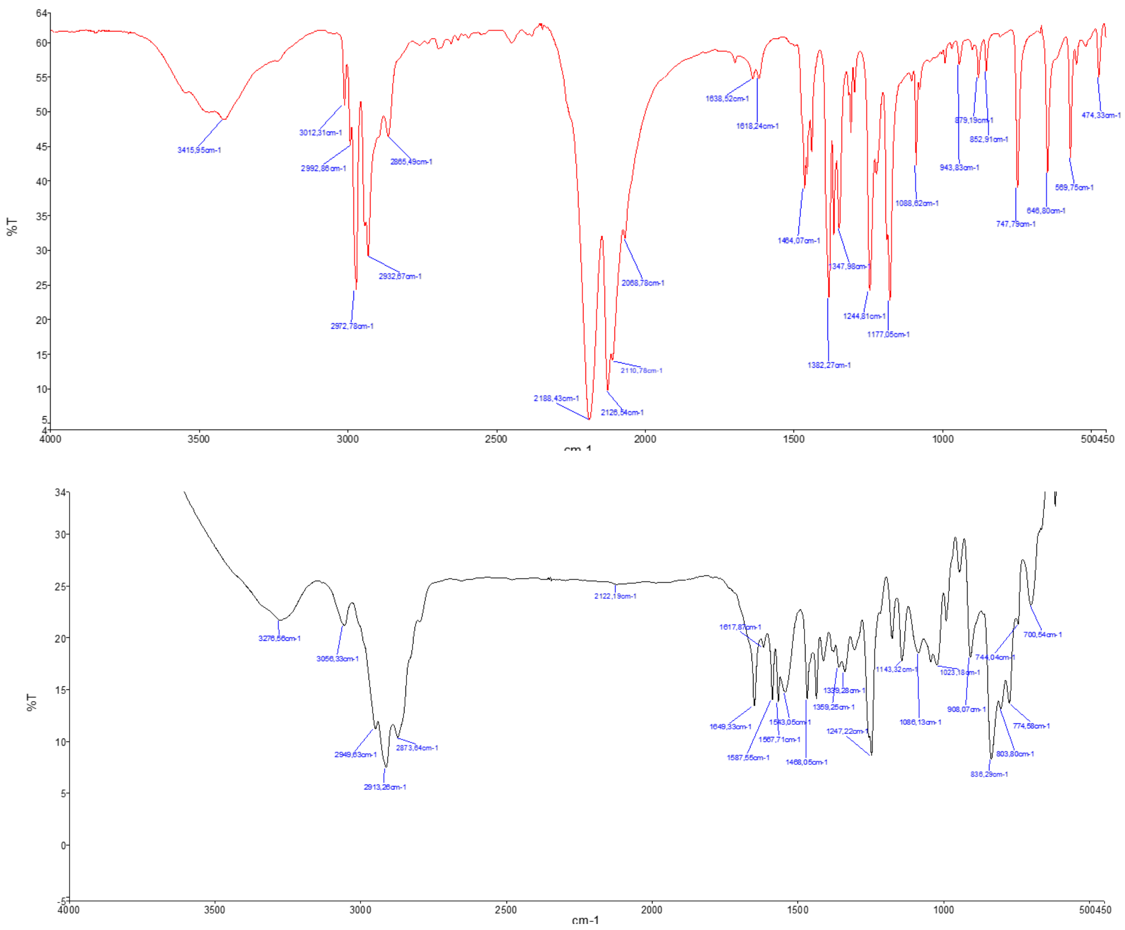

Figure S5. FT-IR spectra of precursor 4-isothiocyanateTEMPO (top) and compound **3** (bottom) in KBr.

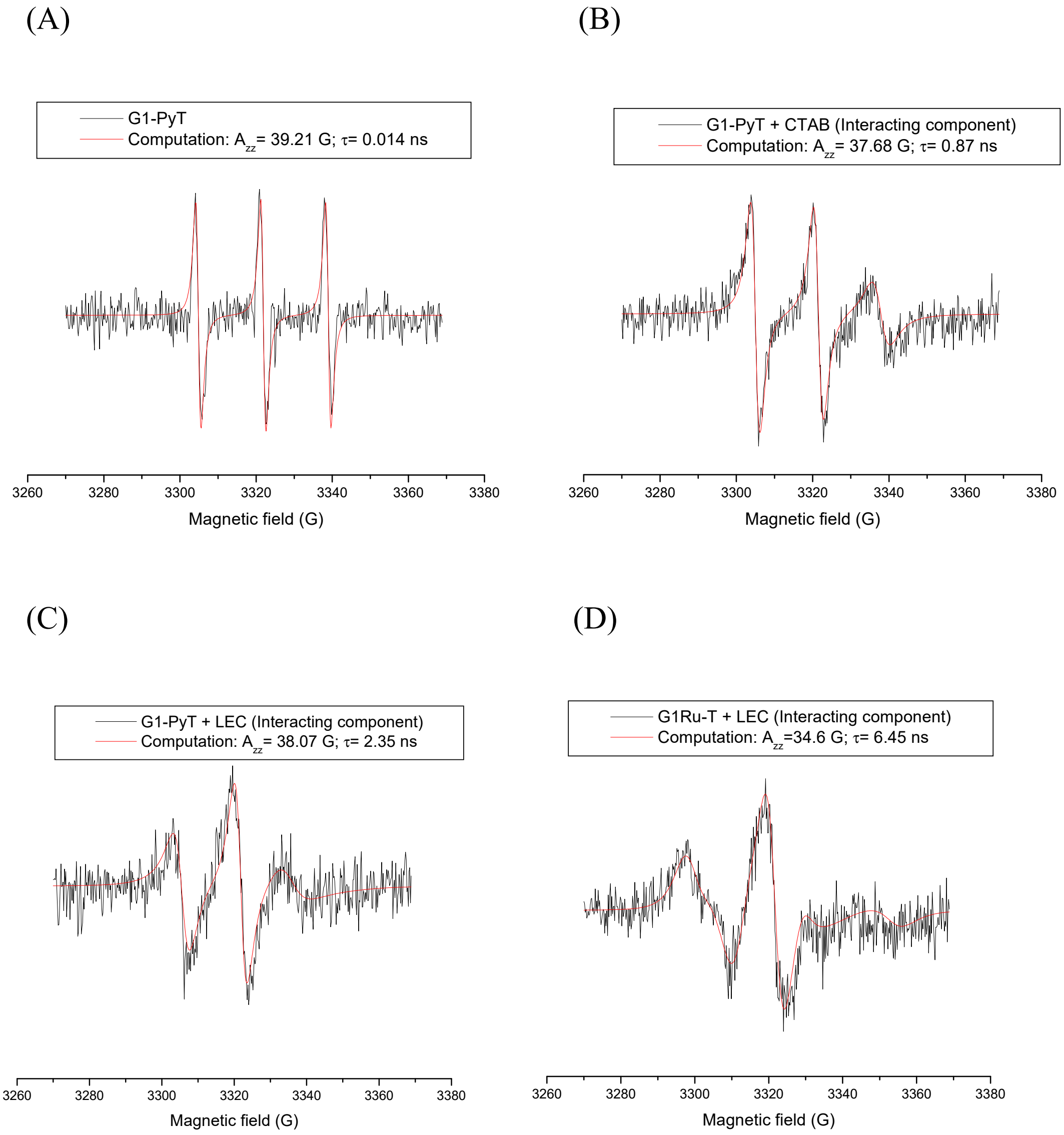

Figure S6. Examples of experimental and computed spectra of labelled dendrimers in the absence and presence of model membranes. Experimental and computed spectra of G_1-PyT, only constituted by the Free component (A). Experimental and computed Interacting component, obtained after subtraction of the Free component, of: (B) G_1-PyT+CTAB; (C) G_1-PyT+LEC; (D) G_1-RuT+LEC. The spectra are normalized in height.

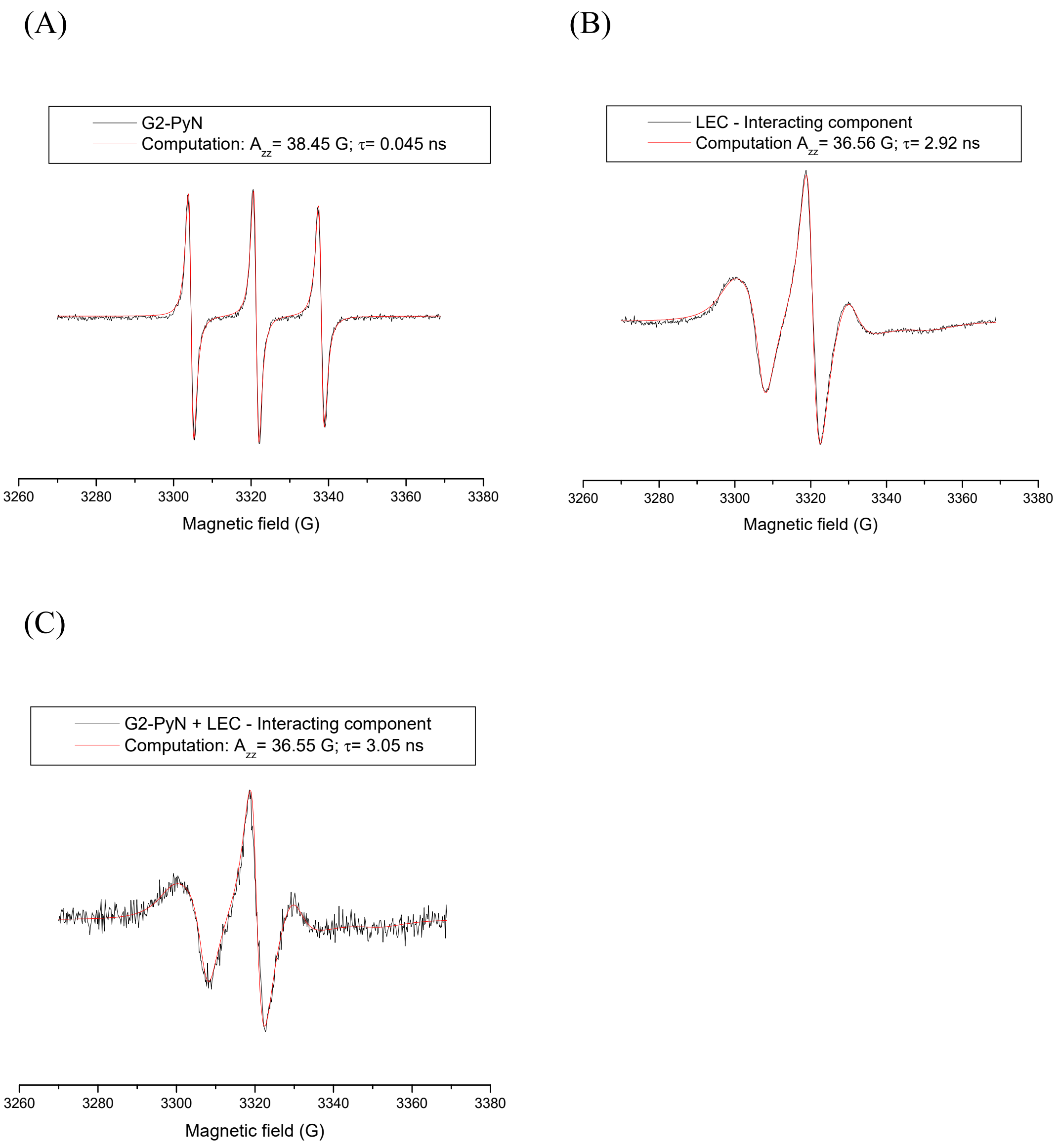

Figure S7. Examples of experimental and computed spectra of non-labelled dendrimers in the absence and presence of model membranes. Experimental and computed spectra of CAT12 in G_2-PyN, only constituted by a free component (A). Examples of computations of the interacting component for CAT12 in: LEC (B); and G_2-PyN+LEC (C).

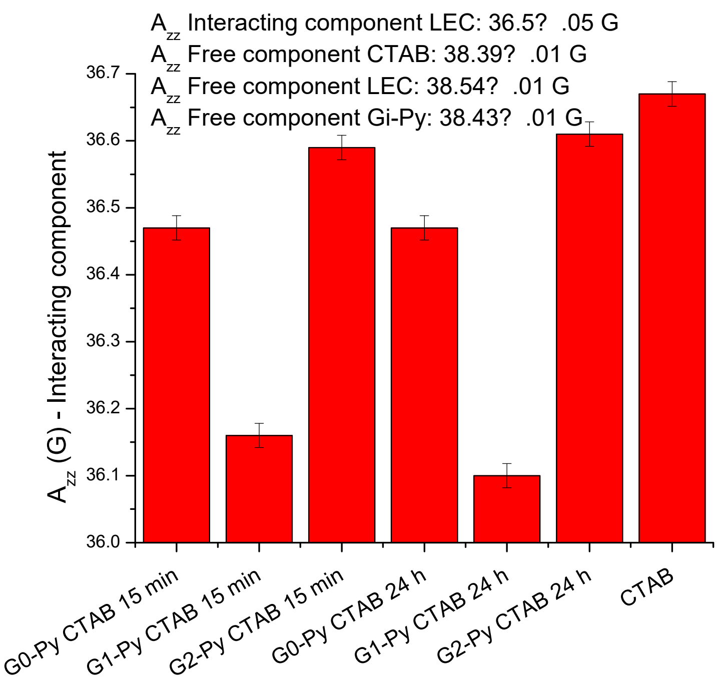

Figure S8: A_{zz} values obtained by computing the Free and the Interacting components for CTAB for homofunctional dendrimers G_n-Py.
